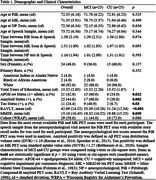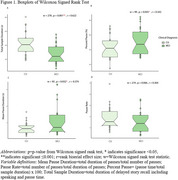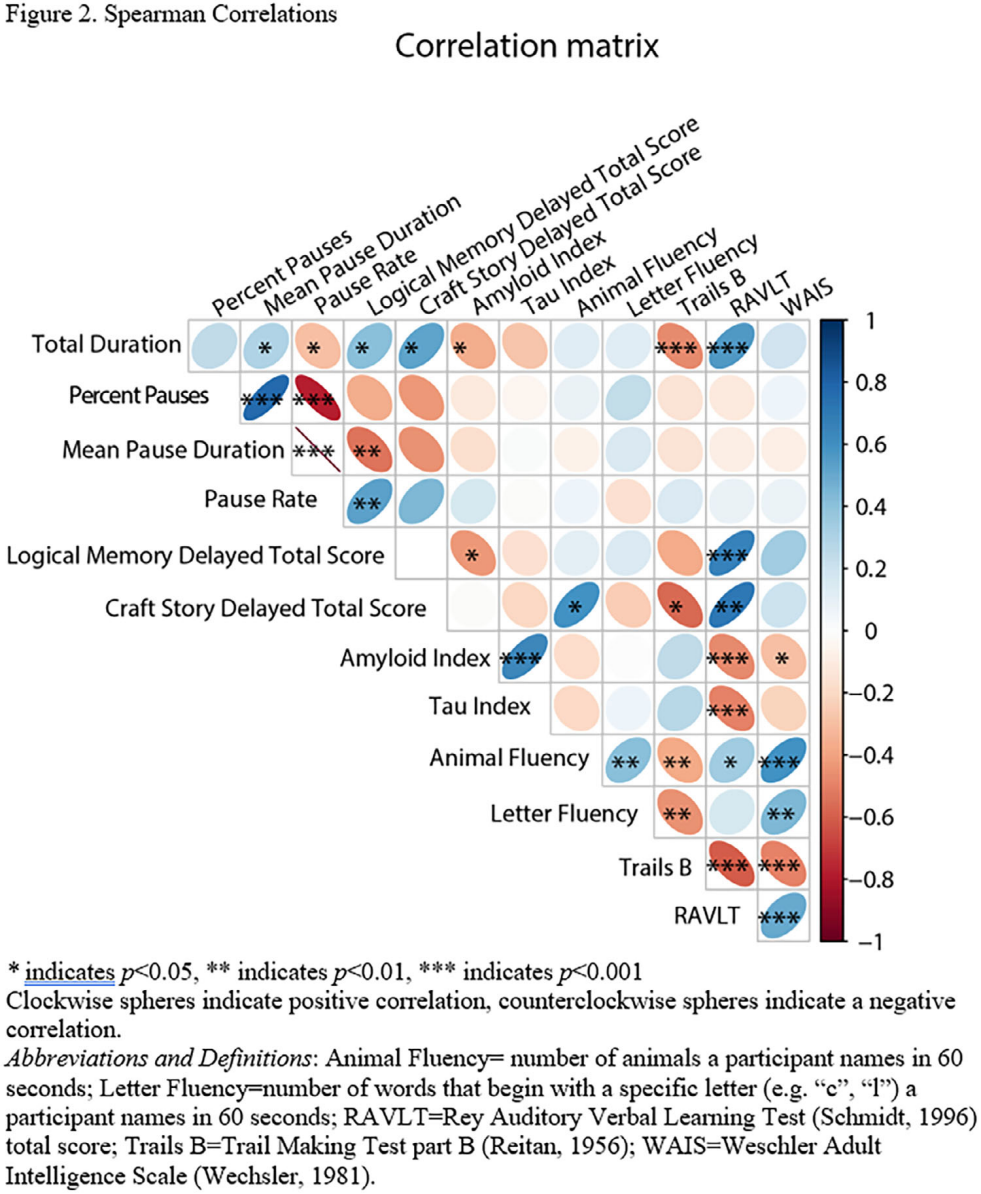# Automated pause analysis of connected speech from story recall as markers of cognitive decline

**DOI:** 10.1002/alz70863_110489

**Published:** 2025-12-23

**Authors:** Madeline R Hale, Deling He, Rebecca E. Langhough, Kristin E Basche, Tobey J. Betthauser, Lingfeng Xu, Julie Liss, Visar Berisha, Bruce P Hermann, Sterling C Johnson, Kimberly D Mueller

**Affiliations:** ^1^ Department of Communication Sciences and Disorders, University of Wisconsin‐Madison, Madison, WI USA; ^2^ University of Wisconsin‐Madison, Madison, WI USA; ^3^ Department of Communication Sciences and Disorders, University of Wisconsin‐ Madison, Madison, WI USA; ^4^ Wisconsin Alzheimer's Disease Research Center, University of Wisconsin School of Medicine and Public Health, Madison, WI USA; ^5^ Department of Medicine, University of Wisconsin‐Madison School of Medicine and Public Health, Madison, WI USA; ^6^ Wisconsin Alzheimer's Institute, University of Wisconsin School of Medicine and Public Health, Madison, WI USA; ^7^ Wisconsin Alzheimer's Institute, University of Wisconsin‐Madison School of Medicine and Public Health, Madison, WI USA; ^8^ Wisconsin Alzheimer's Disease Research Center, University of Wisconsin‐Madison School of Medicine and Public Health, Madison, WI USA; ^9^ Arizona State University, Phoenix, AZ USA; ^10^ Arizona State University, Tempe, AZ USA; ^11^ Department of Neurology, University of Wisconsin‐Madison School of Medicine and Public Health, Madison, WI USA; ^12^ Geriatric Research Education and Clinical Center (GRECC), William S. Middleton Memorial Veterans Hospital, Madison, WI USA; ^13^ Wisconsin Alzheimer's Disease Research Center, School of Medicine and Public Health, University of Wisconsin‐Madison, Madison, WI USA; ^14^ Wisconsin Alzheimer's Disease Research Center, University of Wisconsin‐Madison, School of Medicine and Public Health, Madison, WI USA

## Abstract

**Background:**

Subtle speech features, like pausing, are sensitive to Alzheimer's disease (AD) PET biomarkers. Our previous work showed that silent pauses in Cookie Theft (CT) picture descriptions collected via an acoustic and automatized method differentiated cognitively unimpaired (CU) from cognitively impaired participants The impaired group spoke longer and exhibited longer pauses. The current study examines this pause detection method in story recall (SR).

**Method:**

Fifty participants (*n* = 25 Mild Cognitive Impairment, MCI; *n* = 25 CU) matched by age, sex, race and education using propensity score matching were selected from the Wisconsin Registry for Alzheimer's Prevention (WRAP) and Wisconsin Alzheimer's Disease Research Center (WADRC). Delayed SR audio (Logical Memory (LM) for WRAP, Craft Story (CS) for WADRC) nearest PET scan was cleaned and denoised. Silent pauses >80ms were extracted using MATLAB Speech Pause Analysis. Wilcoxon signed rank tests assessed pause metric differences (effect size: rank‐biserial correlation(r)). Spearman correlation examined pause metrics vs. PET amyloid/tau, neuropsychological tests, and SR scores. CT and SR were compared using logistic regression and ROC analyses.

**Result:**

The mean(sd) years between SR and PET was 1.38(2.19) for amyloid and 1.51(1.89) for tau (Table 1). CU participants had increased SR duration (*p* = 0.001, r=0.622), while the MCI group had longer mean pause duration (*p* = 0.0317, r=0.374) and a higher pause percentage (*p* = 0.045, r=0.342) (Figure 1). No significant difference in pause rate was found. Total duration was correlated with delayed LM (*p* = 0.031, rs=0.417) and CS (*p* = 0.037, rs=0.525) total scores, Trails B (*p* = 0.001, rs=‐0.468), R‐AVLT (*p* = 0.00002, rs=0.566), and amyloid (*p* = 0.011, rs=‐0.357) (Figure 2). Mean pause duration (*p* = 0.004, rs=‐0.537) and pause rate (*p* = 0.004, rs=0.537) were correlated with delayed LM score. Total duration from SR was the only significant predictor of cognitive status (OR=.93, CI=0.89–0.97, AUC=0.76), outperforming CT (OR=1.0, CI=0.99‐1.02, AUC=0.57).

**Conclusion:**

Participants with MCI produced shorter SR samples with a larger percentage and duration of pauses than CU participants. Shorter speech duration correlated with higher amyloid‐beta levels. This contrasts with the longer CT speech duration in the MCI group, possibly due to SR's episodic memory demands. Automated, acoustic‐based pause analysis shows promise as an early AD marker, warranting further study in larger, diverse cohorts.